# Sports Practices and Cardiovascular Risk in Teenagers

**DOI:** 10.5935/abc.20180024

**Published:** 2018-03

**Authors:** Carlos Scherr, Leonardo Corrêa Castro Fabiano, Renata Leborato Guerra, Luciano Herman Juacaba Belém, Ana Carolina Gurgel Câmara, Adriana Campos

**Affiliations:** Fundação Pró Coração - Instituto Nacional de Cardiologia, Rio de Janeiro, RJ - Brazil

**Keywords:** Cardiovascular Diseases / mortality, Risk Factors, Adolescent, Obesity, Hypertension, Exercise, Preventive Health Services

## Abstract

**Background:**

Cardiovascular diseases are the leading cause of deaths in the world, and many events could be prevented by healthy life habits.

**Objectives:**

To compare the occurrence of cardiovascular risk factors in adolescents enrolled at public schools in the city of Rio de Janeiro, including a renowned school for sport practices.

**Methods:**

Cross-sectional study, convenience sampling of 422 students enrolled at the Experimental Olympic Gymnasium (EOG) and at Figueiredo Pimentel School (FP). Using descriptive analyses, continuous variables were expressed as mean and standard deviation or median and interquartile ranges, and the Student's t-test or the chi-square test, respectively, was used for comparisons. The sports were classified according to the metabolic equivalent of task (MET) (below or above 5).

**Results:**

We included 274 students enrolled at the EOG and 148 at FP. Mean age was similar between schools -12.5 ± 1.6 years at FP and 12.6 ± 0.9 at the EOG; 65.5% of the students at FP and 43.8% of the students at the EOG were female (p < 0.01). Significant differences in the prevalence of hypertension (20% vs. 6.3%, p < 0.01) and borderline cholesterol levels (27.7% vs. 17.3%, p = 0.01) were found between FP and EOG students, respectively.

**Conclusion:**

High prevalence of hypertension, overweight/obesity and altered blood lipid profile was found in this group of adolescents. Regular sports training program combined with little influence of their eating habits outside school may contribute to a better metabolic profile and reduction in cardiovascular risk factors in students. Public health measures are also need.

## Introduction

Cardiovascular diseases are the leading cause of death in the world.^1^ It was estimated that 17.5 million people died for cardiovascular diseases in 2012, accounting for 31% of global deaths. More than three-fourths of these deaths were registered in low- and middle-income countries. In addition, 37% of deaths by non-communicable diseases in individuals younger than 70 years are caused by cardiovascular diseases, 3.2 million of them attributed to a sedentary lifestyle.^1^ The majority of cardiovascular diseases may be prevented by strategies aimed at controlling behavioral risk factors, including smoking, unhealthy eating habits and alcohol abuse.^1^

Eating and physical exercise habits acquired during childhood and teenage years may be reflected in adulthood, since evidence indicates that atherosclerosis begins in the first years of life and slowly progresses to adulthood.^2^ In an autopsy study of 100 young individuals who had died from causes unrelated to the cardiovascular system, intimal proliferations were observed in 95.3% of the coronary arterial segments in those aged between one and five years.^3^ In addition, aortic atherosclerosis and lesions in the target organs may be found in hypertensive children.^4^

Studies involving children and adolescents have shown that disturbances of blood pressure and other morphological risk indicators, such as distribution of body fat, may begin during adolescence.^5^ Eating habits and the routine of exercises of the adolescents, developed as they become independent, may potentiate or negatively affect their lifestyle and health in adult age.^6^ It is worth pointing out that childhood is the ideal time to stimulate the practice of regular physical exercise, as this increases the likelihood that this practice will be maintained in adult life. Therefore, the adoption of measures aimed at early prevention of cardiovascular risk factors may enable the primary prevention of heart diseases.^7^

In 2012, the public school network of Rio de Janeiro started a project aimed at integrating academic and sports education - the Experimental Olympics Gymnasium (EOG) - a full-time school focused on sports. Students from the sixth to the ninth grade of elementary school practiced sports for 2 hours, 5 times a week. The exercise program was adequate to each age range group, and followed a long-term athletic development model,^8^ which may contribute to the prevention of future cardiovascular diseases.

The aim of the present study was to evaluate and compare cardiovascular risk factors between adolescent students enrolled in an EOG and students of a public school in which the sports program was not performed.

## Methods

Observational, cross-sectional study conducted with students enrolled in two Rio de Janeiro City public schools. The EOG was located at the district of Santa Teresa. The students were selected for their sports potential to participate in a special training program of different sports, 10 hours weekly, and had five meals a day. The program was started one year before the study. At Fernando Pimentel (FP) School, the students participated in usual physical education activities, one hour per week, and had one meal a day at school.

Students at the sixth to ninth grade of elementary school of both schools were recruited. Students who had the informed consent form signed by their parents or guardians, and who met the criterion of a 12-hour fast before blood collection, were included in the study. The students underwent an interview, physical examination and capillary blood sampling by trained professionals. Blood pressure was measured (in mmHg) with the children comfortably seated, on the right arm, using a calibrated aneroid sphygmomanometer (Welch Allyn Tycos, modelo DS 58-MC). Waist circumference was measured using a measuring tape the midpoint between the iliac crest and the lowest rib. The Accutrend Plus System kit (Roche Diagnostics) was used for determination of glucose, total cholesterol (TC) and triglycerides (TG) levels in the samples of capillary blood. Echocardiography was performed using a Vscan portable ultrasound model 1.0 (GE Healthcare, series number (VH01688751) by a trained technician and all reports were written by a qualified physician. The test results were recorded on the data collection form immediately after the tests were performed.

Each sport was individually analyzed and classified into two categories according to their respective metabolic equivalent of task (MET) (2011 Compendium of Physical Activities) - MET < 5.0/low (table tennis, chess) and MET ≥ 5.0/high (swimming, soccer, judo, athletics, handball and volleyball).^9^ Students who practiced at least one sport with MET ≥ 5.0 were included in the second group. Blood pressure (BP) measurements were categorized according to the percentile of systolic and/or diastolic blood pressure into normal (< 90^th^ percentile), prehypertension (90^th^ - 95^th^ percentile) and hypertension (≥95^th^ percentile).^10^ Capillary blood glucose, and TC and TG levels were classified based on previously published guidelines.^7,11^

### Statistical analysis

Statistical analysis was performed using the Stata software version 12. The Kolmogorov-Smirnov test was used to determine the distribution of continuous variables. Since all continuous variables had a normal distribution, data were expressed by descriptive analysis as mean and standard deviation (SD), and the unpaired t-test was used for comparisons. Categorical variables were expressed as proportion, and the chi-square test used for comparison. Logistic regression was used to assess the association between altered outcomes and exposure variables, with control of all possible confounding variables. Level of significance was set at p < 0.05.

## Results

A total of 148 students enrolled at FP School and 274 enrolled at the EOG were included in the study. Students' mean age was not different between the schools - 12.5 years at FP School and 12.6 years at the EOG. At FP School and EOG, 65.5% and 43.3% of the students, respectively, were female (p < 0.01). Sports practiced at the EOG are described in Table 1. Only 20% of the students participated only in sports classified as MET < 5.0 (table tennis or chess). At FP School, 73.4% of the students did not practice sports regularly outside the school.

**Table 1:** General characteristics of the students enrolled at Fernando Pimentel School (FP) and at the Experimental Olympics Gymnasium (EOG)

		FP (N = 148)		EOG (N = 274)		p value**
		Mean	SD	Mean	SD	
Age		12.5	0.9	12.6	1.6	0.591
		**N**	**%**	**N**	**%**	
Sex	Male	51		154	56.2	< 0.01
	Female	97		120	43.8	
Types of sports*	Low MET (< 5.0)					
	Table tennis	-	-	32	11.4	
	Chess	-	-	25	8.9	
	High MET (> 5.0)					
	Volleyball	-	-	44	15.7	N/A
	Soccer	-	-	41	14.6	
	Handball	-	-	39	13.9	
	Swimming	-	-	36	12.9	
	Athletics	-	-	33	11.8	
	Judo	-	-	29	10.4	
	Unknown	-	-	1	0.4	

SD: standard deviation; MET: metabolic equivalent of task.

* each activity is considered as 1 unit (260 students practiced 1 activity, 4 students practiced 2 activities, and 1 student practiced 3 activities);

** chi-square test (for the categorical variable 'sex') or Student's t-test (for the continuous variable 'age'). N/A: not applicable

Mean weight and body mass index (BMI) were 52.3% kg and 21.2 kg/m^2^ of the students of the FP School and 52.4% kg and 20.7 kg/m^2^ of the students of the EOG (p = 0.28). Mean TC level was 164.3 mg/dL and 158.3 mg/dL at FP School and EOG, respectively, whereas median TG was 89 mg/dL in both schools (interquartile ranges, IQRs: 73-121 mg/dL at FP School and 65-114 mg/dL at the EOG). Mean BP was 110 x 66 mmHg at FP School and 101 x 65 mmHg at the EOG.

Table 2 describes metabolic characteristics of the students. Significant differences were found between the students enrolled at FP School and the EOG in the frequency of SAH (20% *vs*. 6.3%; p < 0.01) and borderline high-cholesterol (27.7% *vs*. 17.3%, p = 0.01). Students from the FP School had a 4.3 times higher chance to develop SAH (odds ratio, OR 4.4; 95% CI 2.1 - 8.6; p < 0.01) and a 1.7 times higher chance to have borderline high TC (OR 1.7; 95% CI 1.05 - 2.8; p = 0.03) than students from the EOG, when both age and sex were considered. Capillary glucose levels were at desirable levels (< 101 mg/dL) in all students from both schools, but 40% of them were overweight or obese. Besides, nearly 50% of the students had TG levels above desirable levels. No difference was found in nutritional status or altered TG between the groups (Table 2).

**Table 2:** Clinical and metabolic characteristics of the students enrolled at Fernando Pimentel School (FP) and at the Experimental Olympics Gymnasium (EOG)

		FP (N=148)		EOG (N=274)		p value*
		N	%	N	%	
Nutritional status (BMI)	Underweight	-	-	2	0.8	
	Normal weight	77	59.2	165	62.0	0.531
	Overweight	33	25.4	64	24.1	
	Obesity	20	15.4	35	13.2	0.567
	Unknown	18	12.2	8	2.9	
Blood pressure	Normal	93	71.5	235	87.0	
	Prehypertension	10	7.7	18	6.7	0.691
	Hypertension	26	20.0	17	6.3	< 0.01
	Unknown	19	12.8	4	1.5	
Capillary blood glucose	Desirable (< 101 mg/dL)	147	100	260	99.6	1.000
	Borderline (101-116 mg/dL)	-	-	1	0.4	
	Increased (≥ 117 mg/dL)	-	-	-	-	
	Unknown	1	0.7	13	4.7	
Total cholesterol	Desirable (< 170 mg/dL)	102	68.9	215	79.0	0.021
	Borderline (170-199 mg/dL)	41	27.7	47	17.3	0.012
	Increased (≥ 200 mg/dL)	5	3.4	10	3.7	0.875
	Unknown	-	-	2	0.7	
Triglycerides	Desirable (< 90 mg/dL)	76	51.4	89	50.3	0.848
	Borderline (90-129 mg/dL)	42	28.4	37	20.9	0.118
	Increased (≥ 130 mg/dL)	30	20.3	51	28.8	0.076
	Unknown	-	-	97	35.4	

Data expressed as absolute values (percentage). BMI: body mass index;

* chi-square test.

Considering the subgroups of EOG students divided by the sports they practiced and respective METs, no differences were found in age or sex between the subgroups. Mean weight in the low MET group was 48.5 ± 13 kg, and 53.3 ± 13.1 kg (p = 0.02); this difference may be ascribed to higher lean mass in the latter. No difference was found in BMI and TC, and the median TG was 89.5 mg/dL in both groups (IQRs: 65-134 mg/dL in low MET and 65-151 mg/dL in the high MET group). Borderline high TC was higher in low MET group than in high MET group (26.8% *vs*. 14.9%; p = 0.04) (Table 3). Considering age and sex, the low MET group had a two times higher chance to have borderline high TC (95% CI 0.98-0.41; p = 0.056).

**Table 3:** Characteristics of the students enrolled at the Experimental Olympics Gymnasium (EOG) by type of sport

		Low MET (N = 56)		High MET (N = 217)		p value*
		Mean	SD	Mean	SD	
Weight (kg)/BMI		48.5	13.0	53.3	13.1	0.02
		20.0	4.8	20.9	4.4	0.20
		**N**	**%**	**N**	**%**	
Nutritional status /BMI	Underweight	-	-	2	0.9	
	Normal weight	37	68.5	127	60.2	0.44
	Overweight	9	16.7	55	26.1	0.15
	Obesity	8	14.8	27	12.8	0.72
	Unknown	2	3.6	6	2.8	
Blood pressure	Normal	48	85.7	186	87.3	0.28
	Prehypertension	6	10.7	12	5.6	0.22
	Hypertension	2	3.6	15	7.0	0.54
	Unknown	-	-	4	1.8	
Capillary blood glucose	Desirable (< 101 mg/dL)	54	98.2	205	100	0.21
	Borderline (101-116 mg/dL)	1	1.2	-	-	
	Increased (≥ 117 mg/dL)	-	-	-	-	
	Unknown	1	1.8	12	5.5	
Total cholesterol	Desirable (< 170 mg/dL)	39	69.6	175	81.4	0.11
	Borderline (170-199 mg/dL)	15	26.8	32	14.9	0.04
	Increased (≥ 200 mg/dL)	2	3.6	8	3.7	
	Unknown	-	-	2	0.9	
Triglycerides	Desirable (< 90 mg/dL)	20	50.0	68	50.0	0.98
	Borderline (90-129 mg/dL)	8	20.0	29	21.3	
	Increased (≥ 130 mg/dL)	12	30.0	39	28.7	
	Unknown	16	28.6	81	37.3	

SD: standard deviation; MET: metabolic equivalent of task; BMI: body mass index

* chi square test (for categorical variables) or Student's t-test (for continuous variables)

Echocardiographic findings were not different between the FP School and the EOG students. Among the EOG students, hypertensive heart disease, interventricular communication and two cases of mitral valve prolapse were identified, whereas in the FP School group, two cases of interventricular communication were detected.

Characteristics of parents/guardians that answered the questionnaire are described in Table 4. Mean age and sex were similar between the two schools - approximately 40 years of age and 85% women. Regular physical activity was more frequently reported by parents/guardians of the students enrolled at the EOG (48% *vs*. 16.5%; p < 0.01), which may have influenced the teenagers to engage in sports. With respect to comorbidities and cardiovascular risk factors, the number of individuals with SAH was 11.2% higher among parents/guardians of the students enrolled at the FP School than at the EOG (30.6% *vs*. 19.4%; p = 0.03).

**Table 4:** Characteristics of the parents/guardians of the students enrolled at Fernando Pimentel School (FP) and at the Experimental Olympics Gymnasium (EOG), who answered the questionnaire

		FP (N = 148)		EOG (N = 274)		p value*
		Mean	SD	Mean	SD	
Age		39,3	8,8	41,3	9,2	0,07
		**N**	**%**	**N**	**%**	
Sex	Male	13	13.1	40	15.9	0.519
	Female	86	86.9	212	84.1	
	Unknown	49	33.1	22	8.0	
Physical activity	No	71	83.5	131	52.0	< 0.01
	Yes	14	16.5	121	48.0	
	Unknown	63	42.6	22	8.0	
Smoking	No	66	77.6	208	82.9	0.26
	Yes	19	22.4	40	15.9	
	Ex-smokers	-	-	3	1.2	
	Unknown	63	42.6	23	8.4	
SAH	No	59	69.4	200	80.6	0.03
	Yes	26	30.6	48	19.4	
	Unknown	63	42.6	26	9.5	
Diabetes	No	79	92.9	240	95.6	0.39
	Yes	6	7.1	11	4.4	
	Unknown	63	42.6	23	8.4	
Previous AMI	No	83	96.5	247	98.4	0.38
	Yes	3	3.5	4	1.6	
	Unknown	62	41.9	23	8.4	
Previous stroke	No	86	100	250	99.6	1.00
	Yes	-	-	1	0.4	
	Unknown	62	41.9	23	8.4	
High cholesterol	No	71	86.6	228	91.6	0.19
	Yes	11	13.4	21	8.4	
	Unknown	66	44.6	25	9.1	

SD: standard deviation; SAH: systemic arterial hypertension; AMI: acute myocardial infarction.

* chi-square test (for categorical variables) or Student's t-test (for the continuous variable 'age')

## Discussion

A high prevalence of cardiovascular risk factors was found in our study group, especially blood lipid levels, overweight / obesity and arterial hypertension. Approximately 50% and 25% of the adolescents had TG and CT concentrations, respectively, above desirable levels (borderline/high); 40% of them were overweight/obese and 17% had prehypertension/hypertension. These data corroborate the current evidence that, despite the importance of malnutrition, the rates of obesity and overweight have been significantly increasing. Previous studies have shown that approximately 23% of children aged between 6 and 12 years and 21% between 12 and 17 years are obese. This increase in obesity prevalence has been attributed to environmental and sociocultural factors.^12^ In a cross-sectional study conducted at schools in Parana State, 154 students aged from 10 to 17 years were assessed for anthropometry, abdominal circumference and BP measurement. The authors reported an association between abdominal obesity and increased BP.^13^

Scherr et al.^14^ reported a significant difference in TC levels between children (mean age 9 years) enrolled at public or philanthropic schools and those enrolled at private schools. In this study,^14^ 23% of boys and girls from private schools and only 4% of boys and girls from public/philanthropic schools had TC levels above 190mg/dL. This may be explained by the intensity of physical exercise and nutritional surveillance in public/philanthropic schools.^14^

The control of cardiovascular risk factors in childhood and adolescence has been recommended worldwide, since several studies have strongly suggested that the presence of risk factors during childhood will affect cardiovascular health in adulthood.^15^ Data of the Bogalusa Heart Study show that excessive adiposity and SAH in childhood and adolescence are associated with myocardial hypertrophy and consequently, higher cardiovascular risk.^16^ In addition, during adolescence, low physical activity level may be associated with higher risk of stroke in the future, whereas participation in physical activity is associated with lower risk for cardiovascular disease, cancer and overall mortality in the future.^17,18^

These results are in agreement with those reported by Crump et al.^19^ from a group of military conscripts at late adolescence, who were followed-up for 43 years. Comparison of the lowest and the highest tertile revealed that high BMI and low aerobic capacity were associated with increased risk of hypertension at adult age.^19^ In the HELENA study, higher levels of cardiorespiratory fitness were associated with a higher number of ideal cardiovascular health components in both boys and girls, especially in boys. These findings in European adolescents indicate that cardiorespiratory fitness, as recommended by the American Heart Association, is positively associated with the ideal cardiovascular health index. Besides, the study identified a hypothetical cardiorespiratory fitness threshold associated with a more favorable cardiovascular health profile, which seems to be more characteristic in boys than girls. Therefore, a lifestyle change focusing on increasing physical activity and improving physical fitness may contribute to the improvement of cardiovascular health.^20^

It is worth mentioning that the association between diet, physical exercise and control of risk factors, with improvement of cardiovascular prognosis, has also been demonstrated in interventional studies. The STRIP (Special Turku Coronary Risk Factor Intervention Project) study followed approximately 530 children from 7 months of age until early adulthood. The intervention group participated in a nutritional counseling program, based on a low-cholesterol, low-saturated fat diet, whereas the control group followed a conventional diet. In the intervention group, there was a significant, favorable impact on the parameters of endothelial function and on reducing cholesterol serum levels.^21^ A study on diabetic adolescents undergoing a physical exercise program showed a better glycemic control and greater reduction in serum lipid levels in those individuals with type 1 diabetes.^22^ In the study by Högström et al.,^23^ healthy Swedish boys at the age of 18 were followed-up for a median period of 34 years. After this time, higher incidence of myocardial infarction was observed in those adolescents with better aerobic fitness as compared with the low fifth of aerobic fitness.^23^

Interestingly, in our study, the practice of regular physical activity was more frequently reported by parents/guardians of the students enrolled at the EOG. These individuals also showed a lower rate of previously diagnosed SAH. It is possible that the attitude of these parents/guardians could have influenced the interest of the students in competitive sports, which corroborates the idea that support and encouragement of parents/guardians for their children to engage in regular physical activity is crucial. A previous study demonstrated that children's healthy behavior in terms of eating habits and physical activity is influenced by parents' behavior, as parents of athletic adolescents used to practice more exercises than those of sedentary adolescents.^24^

### Study limitations

Limitations of the present study included the lack of data on nutritional aspects of these adolescents during periods outside school hours, and how long these students have been engaged in competitive sports (for at least one year). Even greater differences between the groups may have been mitigated by the limited period of competitive sports and the high percentage of missing data on TG in the EOG group. Besides, assessment of nutritional status only by BMI may not be conclusive. However, there is no current consensus on the best BMI classification system to diagnose overweight and obesity in adolescents.^25^ Finally, with respect to our sample, in addition to being adherent to the program, the EOG students came from all parts of the city, and thereby composed a representative sample. On the other hand, the FP group came from a limited number of areas and was composed by convenience sampling, which may represent a limiting factor, since adherence of students who attended school in the afternoon was lower.

## Conclusions

Altered blood pressure, BMI and blood lipid profile were frequent in adolescents enrolled at these public schools. Although more effective public health measures are still required, regular sports training program combined with little influence of their eating habits outside school seem to contribute to a better metabolic profile and reduction in cardiovascular risk factors in students.
